# *Haemonchus contortus* excretory and secretory proteins (HcESPs) suppress functions of goat PBMCs *in vitro*

**DOI:** 10.18632/oncotarget.9589

**Published:** 2016-05-25

**Authors:** Javaid Ali Gadahi, Bu Yongqian, Muhammad Ehsan, Zhen Chao Zhang, Shuai Wang, Ruo Feng Yan, Xiao Kai Song, Li Xin Xu, Xiang Rui Li

**Affiliations:** ^1^ College of Veterinary Medicine, Nanjing Agricultural University, Nanjing, PR China

**Keywords:** Haemonchus contortus, ESP, goat, PBMC, immunomodulation, Immunology and Microbiology Section, Immune response, Immunity

## Abstract

Excretory and secretory products (ESPs) of nematode contain various proteins which are capable of inducing the instigation or depression of the host immune response and are involved in the pathogenesis of the worms. In the present study, *Haemonchus contortus* excretory and secretory products (HcESPs) were collected from the adult worms. Binding of HcESPs to goat peripheral blood mononuclear cells (PBMCs) was confirmed by immune-fluorescence assay. Effects of the HcESPs on cytokine production, cell proliferation, cell migration and nitric oxide (NO) production of PBMCs were checked by co-incubation of HcESPs with goat PBMCs. The results indicated that the production of IL-4 and IFN-γ were significantly decreased by HcESPs in dose dependent manner. On the contrary, the production of IL-10 and IL-17 were increased. Cell migration was significantly enhanced by HcESPs, whereas, HcESPs treatment significantly suppressed the cell proliferation and NO production. These results indicated that the HcESPs played important suppressive regulatory roles on PBMCs and provided highlights to the understanding of the host-parasite interactions.

## INTRODUCTION

Gastrointestinal nematodes generally develop chronic infections and survive themselves in the host for a longer duration. The survival in the host reflects the ability of parasites to evade the host immune responses from the early stages of infection [1, 2]. Excretory and secretory products (ESPs) are produced and released by the parasites during *in vitro* cultivation [3] and *in vivo* [4]. ESPs contain various proteins and are capable to induce the depression or instigation of the host immune response and are related to the pathogenesis of the parasites [5–7]. Binding to the host cell is a prerequisite for ESP function [3, 8–11]. Some ESP molecules react to the molecules on the surface of the host cell to form receptor-ligand complexes, similar to many other receptor-ligand systems. For instance, galectin binds to β-galactoside sugars in a metal-independent manner [12, 13].

Host immune responses are usually inhibited by nematode ESPs through various mechanisms, such as interfering with antigen processing, suppression of macrophage and antigen-presenting cell function, interference with cytokine signaling, or induction of immune-regulatory cell [14, 15]. It was reported that helminth ESPs induced the regulatory T cell (Treg) function in activated CD4^+^ T lymphocytes *in vitro* [15]. Klesius et al [16] showed that O*stertagia ostertagi* could suppress the lymphocyte activation and lymphocyte proliferation during the pre-patent period of infection, whilst, Gomez et al [17] found that ESPs collected from *O*. *ostertagi* L4 were capable to inhibit the mitogen-induced bovine lymphocyte proliferation.

*H. contortus* excretory and secretory products (HcESPs) contain many proteins [18] that can perform various functions including the tissue penetration and host protein degradation [6]. A 55kDa secretory glycoprotein was identified with the ability to inhibit host neutrophils [8]. The purified 66-kDa adult *H. contortus* excretory/secretory (E/S) antigen was confirmed to suppress monocyte function *in vitro* by decreasing the production of hydrogen peroxide and nitric oxide in the culture medium of the cells [3]. Recently, it was reported that recombinant *H. contortus* galectin (rHco-gal-m) inhibited the expression of MHC II molecules, decreased the T cell activation and proliferation, induced apoptosis of T cells and effected several signaling cascades *in vitro* [10]. *In vitro* studies also showed that ESPs had direct effects on cultured cells or tissues, such as inhibiting acid secretion [19] and inducing the vacuolation and detachment of HeLa cells [20, 21]. These findings indicated that ESPs might play multiple functions *in vivo.*

Up to now, some ESP molecules have been recognized and functionally characterized. However, in the natural infection of the nematode, the effects of the ESPs on the host cells and the final roles of the ESPs in the interactions of host and helminths might be dependent on the total ESPs, but not one or several molecules. In the present study, we evaluated the immune-modulatory effects of HcESPs on the goat PBMCs.

## RESULTS

### Confirmation of binding of HcESPs to goat PBMCs

**Figure 1:**
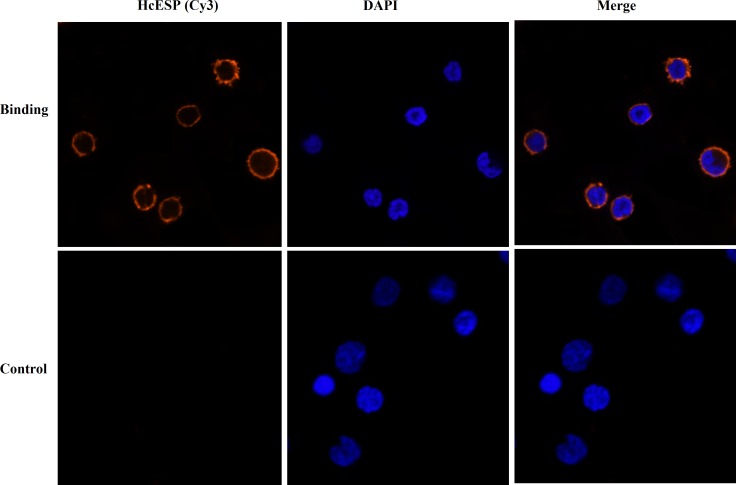
Confirmation of binding of HcESPs to goat PBMCs by IFA The nuclei of the corresponding cells were visualized by DAPI (blue) staining. Staining of the target proteins (red) were visualized by Cy3-conjugated secondary antibody. Merge, overlap of red and blue channels. No red fluorescence was observed in control group.

Goat PBMCs were incubated with HcESPs and the binding was investigated by IFA. The emissions from the Cy3-labeled HcESPs were red and the DAPI-labeled nuclei were blue. Some cells treated with HcESPs showed red fluorescence in the periphery of the blue nucleus. And no fluorescence was observed in the non-treated group. It indicated that HcESPs could bind to the PBMCs (Figure 1).

### Effects of the HcESPs on the production of cytokines of PBMCs

**Figure 2:**
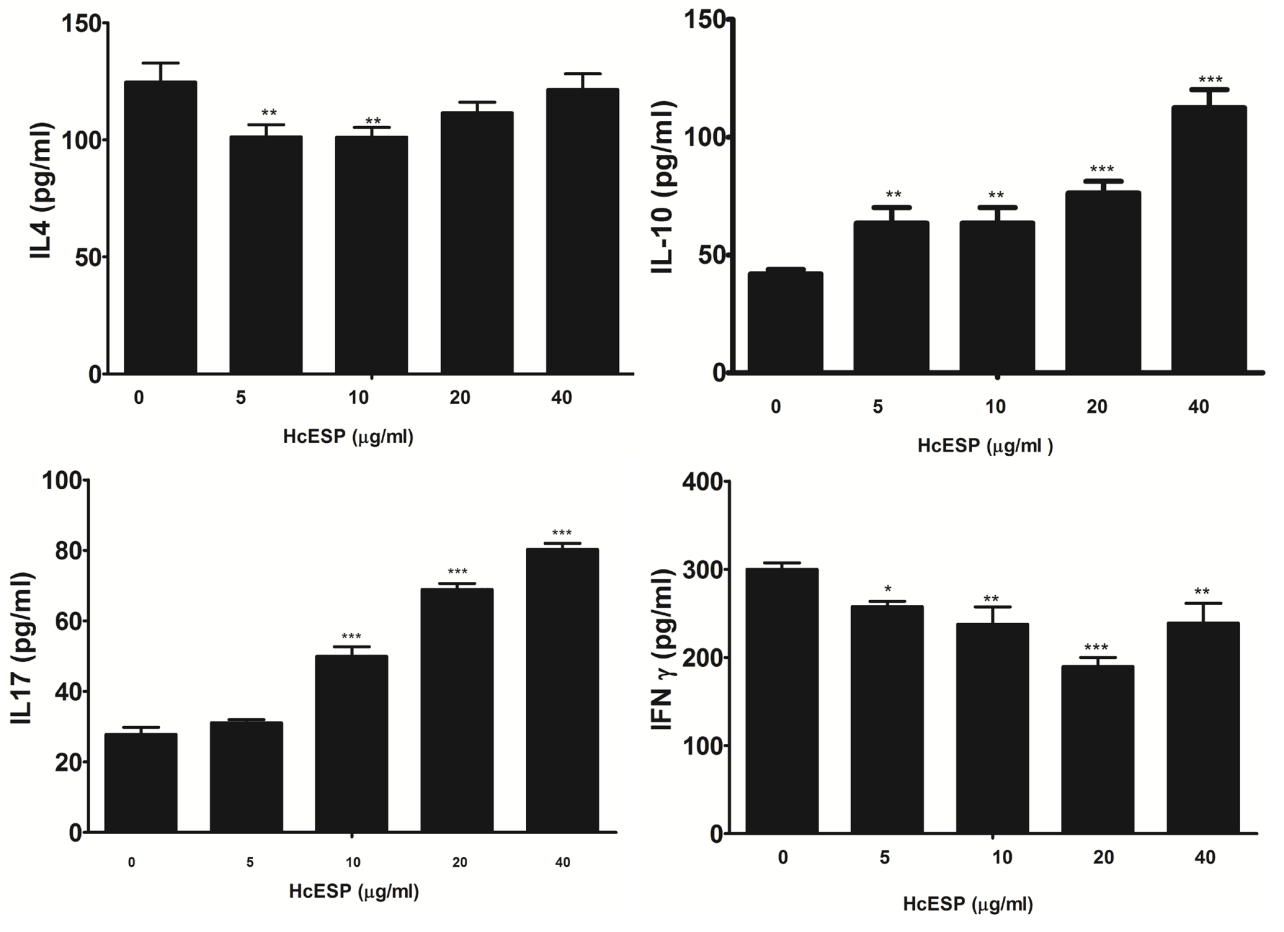
Analysis of the level of multiple cytokine production by PBMCs *in vitro* PBMCs were stimulated with ConA (10 μg/ml) for 24h in the presence or absence of various concentrations of HcESPs. Cytokine secretion in the supernatant of cell cultures was quantified by ELISA. The data are representative of three independent experiments (*p <0.01, **p < 0.001, ns non significant).

Effects of HcESPs on the cytokine production were analyzed by ELISA and the results showed that HcESPs could modulate the cytokine production (Figure 2). Production of IL-4 and IFN-γ were significantly decreased in PBMCs incubated with different concentrations of HcESPs. The productions of IL-4 of 5 μg/ml and10 μg/ml HcESPs treated groups were significantly lower than that of control group. No significant differences were observed between the control and the PBMCs treated with HcESPs at the dose of 20 μg/ml and 40 μg/ml. Various concentrations of HcESPs decreased the IFN-γ levels. Contrary to that, the secretions of cytokine IL-10 and IL-17 were significantly increased by the all concentrations of HcESPs.

**Figure 3:**
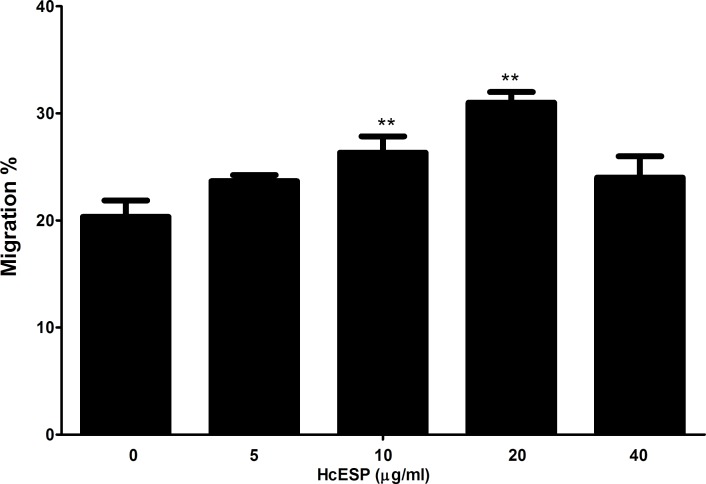
Effects of HcESPs on PBMC migration PBMC were treated with control buffer and various concentrations of HcESPs, Then the random migration was determined. The difference between the mean values was calculated using ANOVA. Data are representative of 3 independent experiments (**p < 0.001 and ***p < 0.0001).

### Cell migration assay

The effect of the HcESPs on the cell migration was evaluated by a Transwell system (Corning, USA). The results showed that 10μg/ml and 20μg/ml of HcESPs significantly increased the cell migration compared to the control, but the 5μg/ml and 40μg/ml not.

### Nitric oxide (NO) production

**Figure 4:**
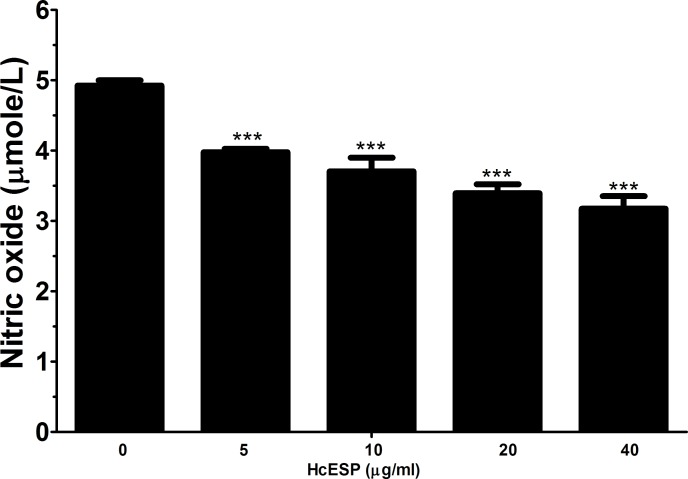
Effects of HcESPs on nitric oxide production by PBMCs *in vitro* Cells s were activated with ConA and incubated at the same time with serial concentrations of HcESPs at 37°C and 5% CO_2_. The nitrite concentration in the PBMCs was measured by using the Griess assay and used as an indicator of nitric oxide production by the PBMCs. The data were representative of three independent experiments (***p < 0.0001).

Nitric oxide (NO) production by PBMCs treated with different concentration of HcESPs was measured by using the total nitric oxide assay kit. The results revealed that HcESPs significantly suppressed the NO production by PBMCs in a dose dependent manner (Figure 4).

### Cell proliferation

**Figure 5:**
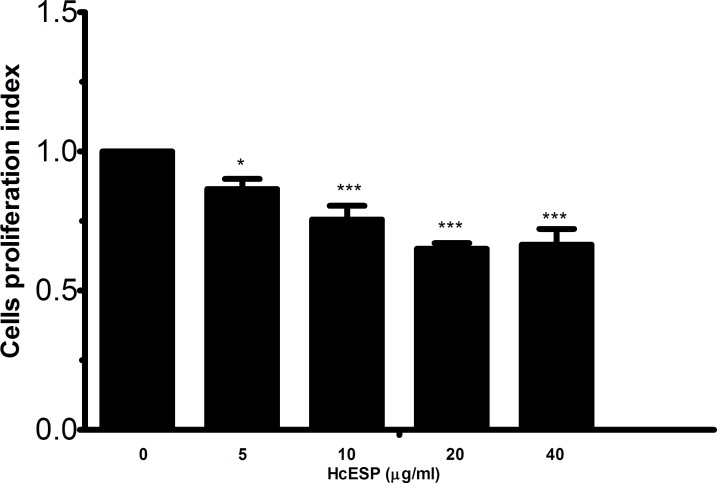
Effects of HcESPs on PBMCs proliferation Cells s were activated with ConA and incubated at the same time with serial concentrations of HcESPs at 37°C and 5% CO_2_. The proliferation was measured by CCK-8 incorporation after 72 h. Cell proliferation index was calculated considering the OD_450_ values in controls as 100%. The data were representative of three independent experiments (*p < 0.01 and ***p < 0.0001.

Cell counting kit (CCK8) was used to evaluate the effects of the HcESPs on the PBMC proliferation. Cell Proliferations were significantly suppressed by all concentrations of HcESPs compared with control group (Figure 5).

## DISCUSSION

Nematode ESPs are molecules secreted by the worms *in vivo* or *in vitro* and played complex functions in the interactions between the parasites and the hosts. However, in the natural infection, the effects of the ESPs on the host and the final roles of the ESPs in the interactions of host and helminths might be dependent on the total ESPs, but not one or several molecules. In the present research, the effects of HcESPs on some functions of goat PBMCs were firstly investigated. The results showed that HcESPs presented suppressive potential on the PBMCs. It showed highlight to the understanding of the interactions of the host and the worms.

It was reported that Type 2 (Th2) and Type 1 (Th1) immune responses and tissue inflammation played important roles in the resistance to the nematodes infections including *H. contortus* [22–24]. Jacobs et al [25] demonstrated that early IL-4 expression was associated with resistance to *Haemonchus contortus* in the resistance sheep breeds. The immune and inflammatory responses were strongly mediated by some cell lines and cytokines they produced. Type 1 and Type 2 responses were positively mediated by Th1 and Th2 cells and their typical cytokines, IFN-γ and IL-4, respectively. Another cell line, T regulatory cells (TReg), and its typical cytokine IL-10 usually played suppressive functions in the immune responses. It inhibited the development of Th2 cell responses [26–28] and decreased the production of IFN-γ [29, 30]. In this study, HcESPs significantly inhibited the productions of IL-4 and IFN-γ, increased the level of IL-10. These results suggested that HcESPs could suppress Type 2 and Type 1 immune responses simultaneously and strengthened the suppression by increasing the production of IL-10. This might be one of the immune evasion mechanisms of *H. contortus*.

Th17 cells and their typical cytokine, IL-17, were functionally characterized as tissue inflammatory modulator [31]. It was demonstrated that neutrophil activation and tissue damage were reduced in IL-17 deficient animals and the increased level of IL-17 was related to the pathogenesis of various parasites [32–36]. In the present research, the IL-17 secretion was significantly increased by HcESPs. This finding suggested that HcESPs could induce the Th17 cells. The enhanced IL-17 level might favour the survival of the worm in host. However, the real relationship of IL-17 and pathogenesis need to be further investigated.

NO was produced by macrophages activated by IFN-*γ* and TNF-*α* and usually played killing functions on the helminths [37, 38]. In the present study, the production of NO was significantly suppressed by HcESPs. This indicated that the HcESPs could inhibit the production of NO through decreasing IFN-*γ* level or other ways, and thus alleviated the harmful effects of some chemical factors produced by the host cells on the helminths.

Cell proliferation and migration are very important to the developments of immune responses. Cell proliferation increased the numbers of the effecter cells and cell migration recruited the effecter cells to the sites of infection. It was reported that helminths actively stimulated eosinophils and other lymphocyte to migrate to the sites of infection and resulted in tissue damage [39, 40]. In this study, it was identified that HcESPs significantly suppressed the goat PBMCs proliferation and significantly enhanced the cell migration. These results showed that HcESPs also inhibited the Type 2 and Type 1 responses through decreasing the numbers of the effecter cells. However, the real roles of the increased cell migration in the immune responses should be investigated.

In our previous studies, we found that recombinant galectin of male and female adult *H. contortus* displayed different effects on the T cells and monocytes [10, 41]. Thus, it was suspected that the ESPs from males and females also had different functions However, in the natural infection of this worm the ESPs from males and females were secreted and functioned simultaneously. So in this research, we used the mixed ESPs from males and females. Thus, differentiating the different effects of ESPs from males and females worms on PBMCs is worth of further investigating. PBMCs are consisted of lymphocytes (T and B), monocytes, DCs and other cells. Perez et al [42] reported that T cells, particularly CD4^+^ and γδ^+^ lymphocytes, in the abomasal mucosa of goats infected with *H. contortus* were mildly increased at 3 and 6 days post infection (dpi) and marked increased at 10 and 13 dpi. B cells and IgG+ plasma cells also showed a marked increase in the abomasal mucosa at 10 and 13 dpi. Whereas the increases in CD8^+^ cells were less pronounced. These results indicated that the infection of *H. contortus* could change the cell populations *in vivo*. In this research, it was demonstrated that HcESPs could effect the functions of the PBMCs of goat. However, the effects of HcESPs on the populations of PBMCs were not studied. Thus, the cell populations that govern the functional changes of PBMCs treated with HcESPs and the protein or proteins that induce the changes *in vitro*, as well as *in vivo*, need to be further probed.

Conclusively, this study identified that the HcESPs displayed suppressive potential on the goat PBMCs *in vitro*. It inhibited the productions of IL-4, IFN-*γ*, increased the suppressive cytokine IL-10, enhanced the inflammatory modulator IL-17, suppressed the production of chemical factor NO, decreased the cell proliferation and activated the cell migration. These results are favorable to the profoundly understanding of the immune invasion and the host-parasite interactions of *H. contortus* and other gastrointestinal nematode. However, for complex life cycle of *H. contortus*, the real effects of the HcESPs on the PBMCs *in vivo* need to be further investigated. Although the functions of some molecules of HcESPs had been characterized, the molecule or the molecules that governed the suppressive functions on the PBMCs also need to be further studied for the huge numbers of the molecules of HcESPs.

## MATERIALS AND METHODS

### Ethics statement

Animal experiments were conducted following the guidelines of the Animal Ethics Committee, Nanjing Agricultural University, China. All experimental protocols were approved by the Science and Technology Agency of Jiangsu Province. The approval ID is SYXK (SU) 2010-0005.

### Collection of adult *H. contortus* worms and *in vitro* production of excretory and secretory products (HcESPs)

Two local male goats (02 years old) were raised under nematode free condition and dewormed twice at 15 days interval by anthelmintic drug. Both goats were orally infected with 10,000 infective stage larvae (L_3_) of *H. contortus*. For the confirmation of the infection, faecal sample was collected and checked after weekly intervals for the presence of *H. contortus* eggs. After confirmation of infection, goats were euthanized and killed at 27^th^ day post infection. The abomasum was tied off at both ends and detached from the remaining digestive tract. Mixed adult worms (male and female) were collected from the abomasums, washed several times in PBS, and kept in RPMI 1640 medium (100 worms/ml) containing 100 IU of penicillin and 0.1 mg/ml streptomycin (Pen strep, gibco, Life Technologies) at 37°C under 5% CO. The parasites were initially incubated for 4 hours, after than the medium was collected and new medium containing 2% glucose was added and incubated for overnight. At the end of incubation period, the cultured media was centrifuged and the supernatant was collected. Collected supernatant was filter-sterilized by using the 0.2 μm pore size membrane filter. HcESP from the collected supernatant was concentrated and desalted (10 mM Tris, NaCl pH7.4) by using the 3-kDa filters (Centripep YM-3, Millipore). The protein concentration was checked by Bradford method (Bradford, 1976).

### Production polyclonal IgG against HcESP (IgGHcESP)

SD Rats were used for the generation of polyclonal antibodies against *H. contortus* excretory and secretory proteins (IgGHcESP). HcESP protein of 400 μg was mixed with Freund's complete adjuvant (1:1) and injected into SD rats subcutaneously [39, 43]. The booster doses were injected four times at 2-week intervals with the same dose to the first immunization and the HcESP was mixed with Freund's incomplete adjuvant at 1:1. One week after the last injection the rats were bled and the sera containing specific anti-HcESP antibodies were collected and stored at −20°C. Blank sera were collected before starting the immunization and kept as negative control.

### Isolation of goat PBMCs and confirmation of HcESP binding to PBMCs

Heparinized blood was collected by vein puncture from dewormed healthy goats. PBMCs were separated with the standard Ficoll-hypaque (GE Healthcare, USA) gradient centrifugation method [44] and washed twice in Ca2+2+/Mg- free PBS pH 7.4. Cell viability assessed by means of the trypan blue exclusion test was consistently >95%. The PBMC were re-suspended to a final density of 1×10^5^ cells/ml in RPMI 1640 medium containing 10% heat inactivated fetal calf serum (FCS), 100 U/ml penicillin and 100 mg/ml streptomycin Pen Strep (Penicillin and Streptomycin) (gibco, Life Technology). PBMCs were incubated in the presence and absence of HcESPs (5μg/ml) for 1 h at 37°C.

Confirmation of binding was determined by an immunofluorescence assay (IFA) as described by Yuan et al [45]. Briefly, washed cells (10^5^/ ml) were fixed with 4% paraformaldehyde on a poly-L-lysine-coated glass slide. The cells were then treated with blocking solution (4% BSA in PBS) for 30 min to minimize background staining. Then cells were incubated with rat anti-HcESP IgG (1:100) as a primary antibody for 2 h. After being washed with PBS for 3 times (5 min each), the cells were incubated with second antibody (1:300) coupled to the fluorescent dye Cy3 (Beyotime, Jiangsu, China) for 1h at room temperature. The nuclear staining was performed with 2-(4-amidinophenyl)-6-indole carbamidinedihydrochloride (DAPI, 1.5 μM; Sigma, MO, USA for 6 min. finally the protein localization was determined by checking the staining patterns with a 100× oil objective lens on a laser scanning confocal microscope (L SM710, Zeiss, Jena, Germany). Digital images were captured using the Zeiss microscope software package ZEN 2012 (Zeiss, Jena, Germany).

### Detection of the cytokine levels by ELISA

Enzyme linked immunosorbent assay (ELISA) was used for the detection of cytokine levels. Briefly, the freshly isolated PBMCs were re-suspended to a final density of 5 × 10^6^ in complete medium (RPMI 1640 supplemented with 100 U/ml penicillin, 100 μg/ml streptomycin, 2 mM L-glutamine, 10% FCS). The cells were activated with ConA (10 μg/ml) and treated at the same time with a serial concentrations of HcESPs (5, 10, 20, and 40 μg/ml) and an equal volume of PBS as control. Then, the cells were seeded into 24-well plates (1ml/well) and cultured for 24h in 5% CO2 atmosphere at 37°C. Next, the plates were then centrifuged at 200 × g for 15 min and the supernatants were collected. The levels of IL-4, IL-10, IL-17 and IFN-γ in supernatants were determined using commercially available goat ELISA kits (Jian chen, China). Three individual experiments were performed.

### Cell migration assay

The cells were placed on the upper layer of a cell permeable membrane and a solution containing the test agent is placed below the cell permeable membrane. Following an incubation period (3-18 hours), the cells that have migrated through the membrane are stained and counted. The membrane is coated with some extracellular matrix component (e.g. collagen) which facilitates both adherence and migration.

The cell migration assay was performed using a Transwell system (Corning, USA), which allows cells to migrate throughout an 8 μm pore size polycarbonate membrane [27]. The treatment group was incubated with different concentrations of HcESPs (5, 10, 20, and 40μg/ml) and the control group was treated with an equal volume of PBS. The cells were placed on the upper chamber of cell permeable membrane and culture media was placed below the membrane. Following the incubation period of 12 hours, the cells migrated to lower chambers were collected and then the random migration was determined. The difference between the mean values was calculated using ANOVA. Each experiment was performed in triplicate.

### Nitric oxide production assay

The goat PBMCs were harvested and washed twice with PBS. Then, 100 μl of cells (1 × 10^6^ cells/ml) were incubated either with PBS and a serial concentrations of HcESPs (5, 10, 20, and 40μg/ml) in 96-well plates in DMEM medium. Production of NO by PBMCs was determine d by measurement of intracellular nitrite in the PBMC by using the Griess assay [46] according to the instructions of Total Nitric Oxide Assay Kit (Beyotime Biotechnology, China). Absorbance of the colored solution at 540 nm (OD540) in each well was measured using a plate reader (Bio-Rad Laboratories, USA). Absorbance values were converted to micromoles per liter (μmol/L) using a standard curve that was generated by addition of 0 to 80 μmol/L sodium nitrite to fresh culture media. Three individual experiments were performed.

### Cell proliferation assay

Cell proliferation assay was performed as described previously [10]. Briefly, PBMCs (1 × 10^6^ cells/ml) were activated with ConA (10 μg/ml) and incubated at the same time with a serial concentrations of HcESPs (5, 10, 20, and 40μg/ml) and an equal volume of PBS as control at 37°C in 5% CO_2_ incubator for 72 h. CCK-8 solutions (Beyotime Biotechnology, China) were added to each well of the plates 4 h before harvesting the cells and the absorbance values at 450 nm (OD_450_) were measured using a microplate reader (Thermo Scientific, USA). Cells exposed to ConA with control buffer served as controls and the OD_450_ in controls were set as 100%. Cell proliferation index was calculated by the formula: OD_450_ rHco-gal-m /OD_450_ control. Each experiment was performed in triplicate.

## References

[1] Maizels RM, Balic A, Gomez-Escobar N, Nair M, Taylor MD, Allen JE (2004). Helminth parasites-masters of regulation. Immunological reviews.

[2] Moreno Y, Gros P-P, Tam M, Segura M, Valanparambil R, Geary TG, Stevenson MM (2011). Proteomic Analysis of Excretory-Secretory Products of Heligmosomoides polygyrus Assessed with Next-Generation Sequencing Transcriptomic Information. PLoS neglected tropical diseases.

[3] Rathore DK, Suchitra S, Saini M, Singh BP, Joshi P (2006). Identification of a 66 kDa Haemonchus contortus excretory/secretory antigen that inhibits host monocytes. Veterinary parasitology.

[4] Schallig HD, van Leeuwen MA, Cornelissen AW (1997). Protective immunity induced by vaccination with two Haemonchus contortus excretory secretory proteins in sheep. Parasite immunology.

[5] Knox DP (2000). Development of vaccines against gastrointestinal nematodes. Parasitology.

[6] Cox GN, Pratt D, Hageman R, Boisvenue RJ (1990). Molecular cloning and primary sequence of a cysteine protease expressed by Haemonchus contortus adult worms. Mol Biochem Parasitol.

[7] Sun Y, Yan R, Muleke CI, Zhao G, Xu l, Li X (2006). Recombinant galectins of Haemonchus contortus parasite induces apoptosis in the peripheral blood lymphocytes of goat. Int J Pept Res Ther.

[8] Anbu KA, Joshi P (2008). Identification of a 55 kDa Haemonchus contortus excretory/secretory glycoprotein as a neutrophil inhibitory factor. Parasite Immunol.

[9] Reinhardt S, Scott I, Simpson HV (2011). Neutrophil and eosinophil chemotactic factors in the excretory/secretory products of sheep abomasal nematode parasites: NCF and ECF in abomasal nematodes. Parasitology research.

[10] Wang W, Wang S, Zhang H, Yuan C, Yan R, Song X, Xu L, Li X (2014). Galectin Hco-gal-m from Haemonchus contortus modulates goat monocytes and T cell function in different patterns. Parasites & vectors.

[11] Wang W, Yuan C, Wang S, Song X, Xu L, Yan R, Hasson IA, Li X (2014). Transcriptional and proteomic analysis reveal recombinant galectins of Haemonchus contortus down-regulated functions of goat PBMC and modulation of several signaling cascades *in vitro*. J Proteomics.

[12] Gitt MA, Wiser MF, Leffler H, Herrmann J, Xia YR, Massa SM, Cooper DN, Lusis AJ, Barondes SH (1995). Sequence and mapping of galectin-5, a beta-galactoside-binding lectin, found in rat erythrocytes. The Journal of biological chemistry.

[13] Kasai K, Hirabayashi J (1996). Galectins: a family of animal lectins that decipher glycocodes. Journal of biochemistry.

[14] Maizels RM, Yazdanbakhsh M (2003). Immune regulation by helminth parasites: cellular and molecular mechanisms. Nature reviews Immunology.

[15] Grainger JR, Smith KA, Hewitson JP, McSorley HJ, Harcus Y, Filbey KJ, Finney CA, Greenwood EJ, Knox DP, Wilson MS, Belkaid Y, Rudensky AY, Maizels RM (2010). Helminth secretions induce de novo T cell Foxp3 expression and regulatory function through the TGF-beta pathway. The Journal of experimental medicine.

[16] Klesius PH, Washburn SM, Ciordia H, Haynes TB, Snider TG (1984). Lymphocyte reactivity to Ostertagia ostertagi L3 antigen in type I ostertagiasis. American journal of veterinary research.

[17] Gomez-Munoz MT, Canals-Caballero A, Almeria S, Pasquali P, Zarlenga DS, Gasbarre LC (2004). Inhibition of bovine T lymphocyte responses by extracts of the stomach worm Ostertagia ostertagi. Veterinary parasitology.

[18] Yatsuda AP, Krijgsveld J, Cornelissen AW, Heck AJ, de Vries E (2003). Comprehensive analysis of the secreted proteins of the parasite Haemonchus contortus reveals extensive sequence variation and differential immune recognition. The Journal of biological chemistry.

[19] Merkelbach P, Scott I, Khalaf S, Simpson HV (2002). Excretory/secretory products of Haemonchus contortus inhibit aminopyrine accumulation by rabbit gastric glands *in vitro*. Veterinary parasitology.

[20] Przemeck S, Huber A, Brown S, Pedley KC, Simpson HV (2005). Excretory/secretory products of sheep abomasal nematode parasites cause vacuolation and increased neutral red uptake by HeLa cells. Parasitology research.

[21] Huber A, Prosl H, Joachim A, Simpson HV, Pedley KC (2005). Effects of excretory/secretory products of Haemonchus contortus on cell vacuolation. Parasitology research.

[22] Babayan S, Attout T, Specht S, Hoerauf A, Snounou G, Renia L, Korenaga M, Bain O, Martin C (2005). Increased early local immune responses and altered worm development in high-dose infections of mice susceptible to the filaria Litomosoides sigmodontis. Medical microbiology and immunology.

[23] Bleay C, Wilkes CP, Paterson S, Viney ME (2007). Density-dependent immune responses against the gastrointestinal nematode Strongyloides ratti. International journal for parasitology.

[24] Lacroux C, Nguyen TH, Andreoletti O, Prevot F, Grisez C, Bergeaud JP, Gruner L, Brunel JC, Francois D, Dorchies P, Jacquiet P (2006). Haemonchus contortus (Nematoda: Trichostrongylidae) infection in lambs elicits an unequivocal Th2 immune response. Veterinary research.

[25] Jacobs JR, Sommers KN, Zajac AM, Notter DR, Bowdridge SA (2016). Early IL-4 gene expression in abomasum is associated with resistance to Haemonchus contortus in hair and wool sheep breeds. Parasite immunology.

[26] Levings MK, Sangregorio R, Galbiati F, Squadrone S, de Waal Malefyt R, Roncarolo MG (2001). IFN-alpha and IL-10 induce the differentiation of human type 1 T regulatory cells. Journal of immunology.

[27] Taylor A, Verhagen J, Blaser K, Akdis M, Akdis CA (2006). Mechanisms of immune suppression by interleukin-10 and transforming growth factor-: the role of T regulatory cells. Immunology.

[28] Grencis RK, Humphreys NE, Bancroft AJ (2014). Immunity to gastrointestinal nematodes: mechanisms and myths. Immunological Reviews.

[29] Ouyang W, Rutz S, Crellin NK, Valdez PA, Hymowitz SG (2011). Regulation and functions of the IL-10 family of cytokines in inflammation and disease. Annual review of immunology.

[30] Xu Y, Chen W, Bian M, Wang X, Sun J, Sun H, Jia F, Liang C, Li X, Zhou X, Huang Y, Yu X (2013). Molecular characterization and immune modulation properties of Clonorchis sinensis-derived RNASET2. Parasites & Vectors.

[31] Song X, Gao H, Qian Y (2014). Th17 differentiation and their pro-inflammation function. Advances in experimental medicine and biology.

[32] Sutherland TE, Logan N, Ruckerl D, Humbles AA, Allan SM, Papayannopoulos V, Stockinger B, Maizels RM, Allen JE (2014). Chitinase-like proteins promote IL-17-mediated neutrophilia in a tradeoff between nematode killing and host damage. Nat Immunol.

[33] da Matta Guedes PM, Gutierrez FR, Maia FL, Milanezi CM, Silva GK, Pavanelli WR, Silva JS (2010). IL-17 produced during Trypanosoma cruzi infection plays a central role in regulating parasite-induced myocarditis. PLoS neglected tropical diseases.

[34] Fu Y, Wang W, Tong J, Pan Q, Long Y, Qian W, Hou X (2009). Th17: a new participant in gut dysfunction in mice infected with Trichinella spiralis. Mediators of inflammation.

[35] Larkin BM, Smith PM, Ponichtera HE, Shainheit MG, Rutitzky LI, Stadecker MJ (2012). Induction and regulation of pathogenic Th17 cell responses in schistosomiasis. Seminars in immunopathology.

[36] Mbow M, Larkin BM, Meurs L, Wammes LJ, de Jong SE, Labuda LA, Camara M, Smits HH, Polman K, Dieye TN, Mboup S, Stadecker MJ, Yazdanbakhsh M (2013). T-helper 17 cells are associated with pathology in human schistosomiasis. The Journal of infectious diseases.

[37] Cervi L, Rossi G, Cejas H, Masih DT (1998). Fasciola hepatica-induced immune suppression of spleen mononuclear cell proliferation: role of nitric oxide. Clinical immunology and immunopathology.

[38] Gazzinelli RT, Oswald IP, James SL, Sher A (1992). IL-10 inhibits parasite killing and nitrogen oxide production by IFN-gamma-activated macrophages. Journal of immunology.

[39] Wang W, Yuan C, Wang S, Song X, Xu L, Yan R, Hasson IA, Li X (2014). Transcriptional and proteomic analysis reveal recombinant galectins of Haemonchus contortus down-regulated functions of goat PBMC and modulation of several signaling cascades *in vitro*. Journal of proteomics.

[40] Turner DG, Wildblood LA, Inglis NF, Jones DG (2008). Characterization of a galectin-like activity from the parasitic nematode, Haemonchus contortus, which modulates ovine eosinophil migration *in vitro*. Veterinary immunology and immunopathology.

[41] Yuan C, Zhang H, Wang W, Li Y, Yan R, Xu L, Song X, Li X (2015). Transmembrane protein 63A is a partner protein of Haemonchus contortus galectin in the regulation of goat peripheral blood mononuclear cells. Parasites & vectors.

[42] Perez J, Zafra R, Buffoni L, Hernandez S, Camara S, Martinez-Moreno A (2008). Cellular phenotypes in the abomasal mucosa and abomasal lymph nodes of goats infected with Haemonchus contortus. Journal of comparative pathology.

[43] Han K, Xu L, Yan R, Song X, Li X (2012). Molecular cloning, expression and characterization of enolase from adult Haemonchus contortus. Research in veterinary science.

[44] Wang Y, Yang W, Cama V, Wang L, Cabrera L, Ortega Y, Bern C, Feng Y, Gilman R, Xiao L (2014). Population genetics of Cryptosporidium meleagridis in humans and birds: evidence for cross-species transmission. International journal for parasitology.

[45] Yuan C, Zhang H, Wang W, Li Y, Yan R, Xu L, Song X, Li X (2015). Transmembrane protein 63A is a partner protein of Haemonchus contortus galectin in the regulation of goat peripheral blood mononuclear cells. Parasit Vectors.

[46] Green LC, Wagner DA, Glogowski J, Skipper PL, Wishnok JS, Tannenbaum SR (1982). Analysis of nitrate, nitrite, and [15N]nitrate in biological fluids. Analytical biochemistry.

